# A Case Series of Adult-Onset Rasmussen’s Encephalitis: Diagnostic and Therapeutic Challenges

**DOI:** 10.3389/fneur.2017.00564

**Published:** 2017-10-25

**Authors:** James Francis Castellano, Jenny A Meyer, Fred Alexander Lado

**Affiliations:** ^1^Saul Korey Department of Neurology, Montefiore Medical Center, Bronx, NY, United States; ^2^Epilepsy Division, Northwell Health, Manhasset, NY, United States

**Keywords:** autoimmune epilepsy, Rasmussen’s encephalitis, epilepsy, electroencephalography, magnetic resonance imaging

## Abstract

Rasmussen’s encephalitis (RE) is a rare neurologic disorder characterized by progressive cerebral hemiatrophy and medically refractory epilepsy. The majority of current literature on this topic is focused on the pediatric population. In this case series, we will review three cases of adult-onset RE, as defined by fulfillment of the 2005 Bien criteria. The diagnostic challenge of characterizing this rare disease will be highlighted by the extensive serum, CSF, and pathologic sampling in all three patients. MR imaging and EEG data will be examined over time to characterize hallmark findings as well as progression. In addition, we will review the various forms of therapy attempted in these three patients, namely anti-epileptic drug therapy and immunomodulatory therapy. We will also utilize this case series to critically evaluate the broader context of atypical presentations of this disease and the value of current diagnostic criteria.

## Introduction

Rasmussen first described his eponymous disease in 1958 as a progressive epileptic disorder in children due to chronic unilateral encephalitis (1). While these two core clinical characteristics have remained disease hallmarks, we now recognize that Rasmussen’s encephalitis (RE) is not exclusively a childhood pathology. There are multiple reports of adult-onset RE (2–17). In fact, the revised Bien criteria now no longer contain age at onset as a diagnostic criterion (18). While the pathophysiology of this progressive disease remains unknown, current thinking points toward an immune-mediated mechanism. This hypothesis is based on pathologic specimens as well as variable clinical responses to immunomodulatory therapy. The indolent course of this disease in adult populations, coupled with its relative rarity, poses a diagnostic challenge to clinicians. Diagnosis, however, is not the only clinical obstacle. To date, there is no definitive consensus on treatment, with proposed strategies ranging from acute and chronic immunotherapy to hemispherectomy. Our case series of three adult-onset cases of RE exemplify the diagnostic and therapeutic challenges.

### Case 1

In July 2010, a 53-year-old woman with no significant neurologic history experienced a single convulsion. Four months later, she experienced two similar convulsions, quickly progressing to status epilepticus. She was treated at an outside institution and recovered full function and returned to work. Initial MR imaging at that time was reported as normal. However, despite AED treatment, she continued to have focal seizures, characterized by clonic jerks of the right face. Over the next year, her seizures progressed in both frequency as well as semiology, involving the right arm as well as language. Admission to an outside epilepsy monitoring unit (EMU) revealed both electroclinical (semiology of right face/arm movements) and purely electrographic seizures arising from the left frontal region in the context of minimal responsiveness, consistent with focal status epilepticus. She was again treated for status epilepticus and discharged on AED polytherapy: phenytoin, valproic acid, levetiracetam, and oxcarbazepine.

**Figure 1:**
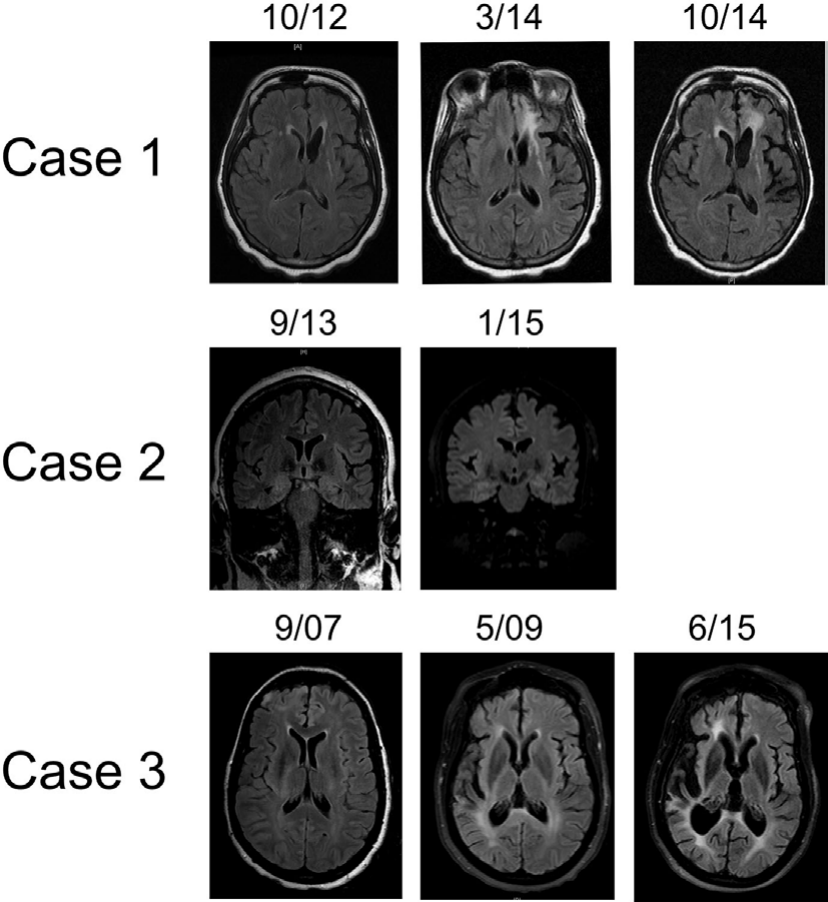
Representative T2 FLAIR MR images highlighting hemispheric atrophy over time. Patient 1 (top panel) showed left frontal predominant atrophy over a 2-year period; axial sections. Patient 2 (middle panel) displayed focal left frontal atrophy over an 18-month period; coronal sections. Patient 3 (bottom panel) had profound right cortical atrophy over a 7-year period; axial sections. MR images were obtained on machines using a 3-T magnet.

As her epilepsy became more refractory, a very extensive etiologic work-up was performed at an outside institution, approximately 2 years from epilepsy onset. MRI demonstrated a left parietal FLAIR hyperintensity with mild associated volume loss (Figure 1). CT imaging of the chest, abdomen, and pelvis did not reveal malignancy. Extensive serum and CSF studies, notably paraneoplastic and autoimmune encephalitis antibody testing (including AMPA-R, GABA B-R, ANNA-1, ANNA-2, ANNA-3, AGNA-1, PCA-1, PCA-2, PCA-Tr, amphiphysin, CRMP-5, VGKC, NMDA-R) as well as genetic testing for CADASIL and MELAS were also negative.

**Figure 2:**
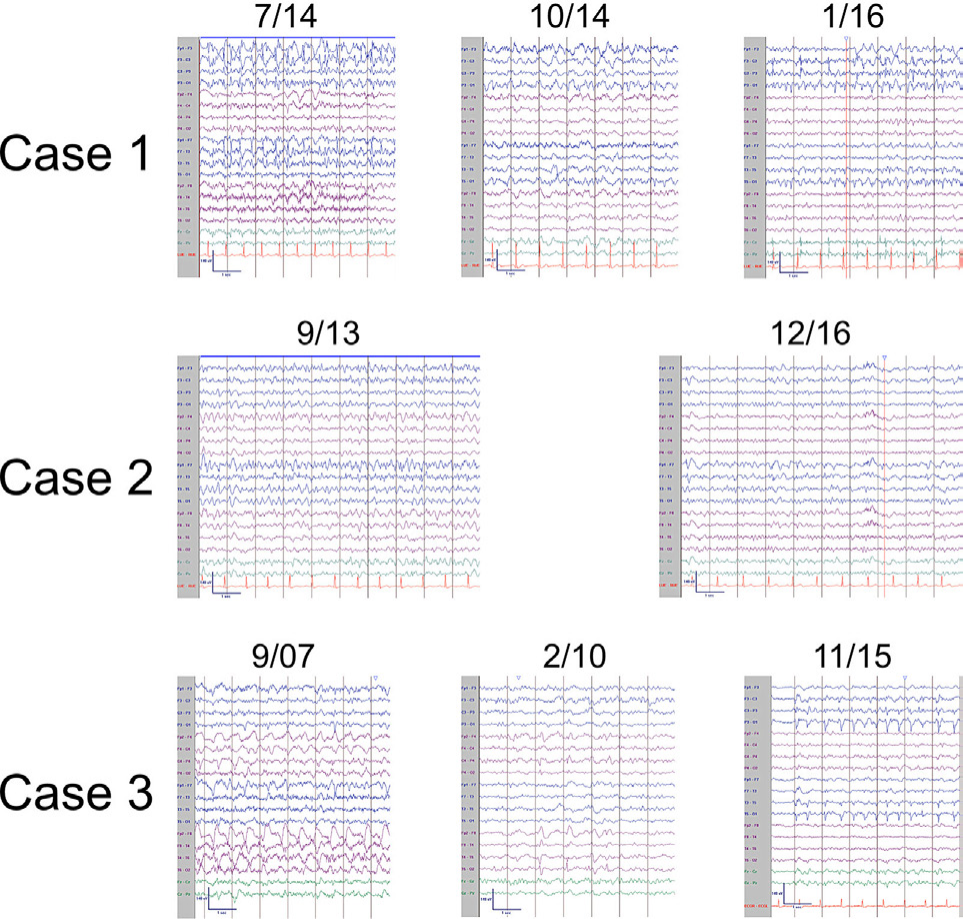
Representative ictal and interictal EEGs. Patient 1 (top panel) displayed left frontal seizures with accompanying right arm and face twitching consistent with epilepsia partialis continua (7/14) and focal interictal slowing (10/14). Later, left occipital seizures (1/16) were also observed. Patient 2 (middle panel) also had left frontal seizures (9/13) and focal interictal slowing (12/16). Patient 3 (bottom panel) had right hemispheric seizures (9/07) with focal interictal slowing (2/10) early in the disease course and developed left sided seizures (11/15) in later stages. EEGs arranged in longitudinal bipolar configuration with sensitivity of 7 μV/mm.

In addition to a known left frontoparietal epileptic focus, the patient then developed a second focus in the ipsilateral occipital quadrant, first observed in May 2013 (Figure 2). Brain PET imaging performed showed overall left hemisphere hypometabolism, with areas of increased metabolism in the left frontal, parietal, and occipital regions, thought to represent ictal changes. Treatment broadened to include rescue doses of benzodiazepines and implantation of a vagal nerve stimulator, approximately 3 years from epilepsy onset. After whole body FDG PET imaging did not reveal occult malignancy, an autoimmune etiology of her epilepsy was presumed and immune therapy was initiated. Single trials of high dose intravenous methylprednisolone (1000 mg × 5 days, March 2014), plasma exchange treatments (5 treatments over 10-day period, May 2015), as well as an induction dose of Rituximab (1000 mg, May 2015) had no clinical effect. During these immunotherapy trials, the patient also underwent brain biopsy targeting the left frontal lobe. The biopsy showed only mild gliosis and rare T lymphocytes, without evidence of vasculitis, microglial nodules, or viral cytopathic changes. Concurrent AED therapy included trials of vigabatrin, zonisamide, and clobazam, with only the last having a meaningful impact on seizure frequency, albeit at a high dose (40 mg daily).

At this point, approximately 4 years from first clinical manifestation, the patient presented to our institution. At the time, the patient was having roughly 20 clinical seizures per day and her AED regimen was as follows: lacosamide, levetiracetam, carbamazepine, zonisamide, clonazepam, and clobazam. Her clinical exam was notable for a moderate mixed aphasia and a mild right hemiparesis. She was admitted to the EMU and found to be in epilepsia partialis continua with near continuous right face and arm twitching. After loading doses of levetiracetam and fosphenytoin, the patient began having distinct episodes of right arm and face movements coupled with mutism. The electrical correlate on EEG monitoring was frequent, brief (average duration of 30 s) left frontal seizures (Figure 2). AEDS were further adjusted, with the addition of perampanel and prn dosing of lorazepam having the greatest effect on seizure frequency reduction. Based on progressive hemiatrophy on imaging (Figure 1), medically refractory epilepsy, static neurologic deficits (right hemiparesis and mixed aphasia), and the extensive serum and CSF sampling, the diagnoses of RE, while conjectured in the past, was finally established. The patient was treated with regular dosing of IVIG. A dose interval of 3 weeks was determined based on the patient family’s impression of when seizure frequency began to increase after the last dose of IVIG. Despite regular IVIG dosing for approximately 6 months as well as concomitant AED therapy, the patient’s epilepsy remained refractory. Currently, the patient is under the care of another institution and IVIG therapy has been discontinued at the behest of the family and her AED regimen was adjusted to include felbamate, lacosamide, levetiracetam, phenobarbital, and lorazepam. Presently, she has a dense right hemiparesis and severe global aphasia.

### Case 2

In 2001, a 44-year-old woman began having focal aware seizures characterized by episodic expressive aphasia. MRI at that time did not reveal abnormalities and her seizures were well controlled with AED polytherapy. Repeat MRI 1 year later revealed a small left frontal lobe T2 hyperintensity without enhancement. Her seizures remained well controlled over the next 10 years on a combination of oxcarbazepine, topiramate, and levetiracetam, and she continued working. Approximately 11 years after first lifetime seizure, she experienced an episode of complete expressive aphasia lasting hours and presented to our institution. She was diagnosed with focal status epilepticus, characterized clinically by expressive aphasia. EEG findings were significant for electroclinical seizures with left frontal onset as well as continuous spike/wave activity in the left frontal region (Figure 2). MRI at that time showed swelling of the left cortical frontal lobe and operculum gray matter as well as mild left frontal atrophy (Figure 1). Initial serum and CSF studies, including cell counts were normal. Further testing was notable for the following negative studies: paraneoplastic and autoimmune encephalitis antibody (NMDA-R, ANNA-1, ANNA-2, ANNA-3, AGNA-1, PCA-1, PCA-2, PCA-Tr, Amphiphysin, CRMP-5, VGKC, GAD), 14-3-3, serum lyme, NYS viral encephalitis panel, ANA, ACE. The patient was acutely treated with phenobarbital, which abolished the status epilepticus. IVIG was also initiated over this hospitalization for the possibility of an autoimmune etiology without specific consideration of RE. She was discharged from the hospital on four AEDs—oxcarbazepine, topiramate, levetiracetam, and phenobarbital. Subsequent clinical improvement over the next several months, initially thought to be related to phenobarbital, waned, leading to the conclusion that IVIG had been the ameliorative therapy. The patient began monthly IVIG infusions to supplement AED pharmacotherapy and experienced improvement in expressive aphasia and a decline in seizure frequency.

Approximately 6 months later, the semiology of the patient’s seizure again evolved to include right rhythmic facial twitching in addition to expressive aphasia. Less than 1 year from prior episode of status epilepticus, she was re-admitted to the EMU. There was no evidence of electrographic seizures, but the presence of left frontal spike/wave complexes persisted as did episodes of right facial twitching and mutism. Ictal SPECT imaging revealed a focus of increased uptake in the left anterior frontal lobe, relative to the right frontal lobe, correlating with MRI abnormality. Over the following months, patient’s aphasia became nearly constant and semiology again began to involve the right arm. Repeat MR imaging now very clearly showed focal atrophy in the left frontal lobe, particularly involving the frontal pole, as well as the left temporal lobe (Figure 1). This finding coupled with the progressive nature of the patient’s focal epilepsy solidified the diagnosis of RE. Monthly IVIG treatments were continued, topiramate does was augmented, and PRN lorazepam was added to the patient’s AED regimen. Unfortunately, the patient continued to have brief focal seizures several times per week and her mild expressive aphasia was static. In September 2016, approximately 3 years after her presentation to our institution and 15 years from first seizure, she began treatment with Rituximab. While her overall seizure frequency appears to have improved, she continues to have weekly focal seizures, a static mild expressive aphasia and remains on four AEDs.

### Case 3

In March 2007, a 47-year-old woman with a history of bilateral optic neuritis at age 35 experienced her first seizure. One month later, she experienced a second seizure and was started on lamotrigine. She presented to our institution as an elective admission to the EMU 6 months after seizure onset. Many right temporal seizures (clinical semiology of paresthesias over left face and arm) were observed and the interictal EEG was notable for right periodic lateralized discharges (PLDs; Figure 1). MRI showed abnormal T2 signal in the right frontal cortex, right thalamus as well as a small area of contrast enhancement in the right temporal uncus. MR SPECT showed normal choline:creatinine ratio. Serum studies were notable for the following normal/negative studies: ANA, ACE, anti-TPO, Lyme, paraneoplastic antibody (ANNA-1, ANNA-2, ANNA-3, AGNA-1, PCA-1, PCA-2, PCA-Tr, CRMP-5, VGKC). CSF evaluation was significant for a mild lymphocytic pleocytosis, negative HSV and CMV PCR, and cytology negative for malignant cells. Ultimately, after trials of several AEDs including lamotrigine, levetiracetam, and carbamazepine, oxcarbazepine and phenytoin were utilized for seizure control.

Over the next 3 years, the patient had several admissions to our institution for breakthrough seizures. Her seizure semiologies over this time frame were isolated left face and arm paresthesias, focal motor seizures involving left face and arm as well as secondarily generalized tonic/clonic seizures. A static mild left hemiparesis, most affecting the left hand, was also noted during this time. Further diagnostic work-up over one of these later admissions was notable for normal CSF cell counts, a CT scan of the chest, abdomen, and pelvis negative for malignancy, and repeat negative paraneoplastic panel evaluation. At this point, the patient was lost to follow-up.

She re-presented to our institution 5 years later after she experienced a generalized convulsion. Collateral history indicated that she had become unable to live at home unassisted and often had confusional and agitated episodes. Since last seen at our institution, her AED regimen had been changed to levetiracetam and valproic acid. In addition, a third lumbar puncture was performed and notably NMDA-R antibody testing was negative. The patient was found to be in non-convulsive status epilepticus and treated with benzodiazepines and augmented doses of her AEDs. CT imaging done on this admission revealed marked right hemisphere atrophy. Continuous EEG monitoring showed both right frontal and temporal spikes and left temporal slowing. Clinical exam was significant for perseverative speech, inability to follow complex commands and a moderate left hemiparesis. Given the patient’s marked hemiatrophy, refractory epilepsy, and static neurologic deficits, she was diagnosed with RE and monthly IVIG was initiated.

MR imaging performed showed marked right hemisphere volume loss, affecting both gray and white mater (Figure 1). Despite the marked asymmetry on imaging findings, EEG monitoring during this advanced stage of disease revealed bihemispheric electrographic pathology; in addition to PLDs in the right posterior quadrant, PLDs and spikes were observed over the left temporal region as well as bilateral independent frontal spikes (Figure 2). By family and caregiver report, patient’s overall seizure frequency decreased over the 5-month period she was treated with immunotherapy. As there was no significant change in the patient’s overall neurologic functional status, decision was made by family to discontinue IVIG infusions and pursue hospice.

Written informed consent was obtained from patients or next of kin for publication of these three case reports.

## Discussion

Taken together with the available literature, this case series of adult-onset RE not only highlights the core clinical characteristics, but also directs attention to many misconceptions surrounding this neurodegenerative process. While the diagnosis of RE has certainly evolved from that initial eponymous paper, the boundaries of this condition remain poorly defined. We will utilize the diversity of this case series as well as numerous examples from the literature to critically evaluate the modern concept of RE, including current diagnostic criteria (18).

We begin our discussion by examining the natural clinical history. There are three well-documented phases of the disease: a prodromal phase with an intermediate frequency of focal seizures and no static neurologic deficits, an acute phase marked by more frequent seizures, hemispheric volume loss, and deficits attributable to that atrophy, and lastly a residual phase with fewer seizures, marked hemiatrophy and severe neurologic deficits (19). Though our three patients all followed this paradigm, both duration and severity were variable (Table 1). Concordant with the other cases of adult-onset RE (2, 3, 7, 8, 13, 14, 20), our three patients had long prodromal and acute phases (Table 1) and a very ill-defined residual period. This is distinct from pediatric populations, in which the average length of the prodromal period has been described as 7.1 months (18). This extended prodromal period makes early diagnosis very challenging. Furthermore, given the continued discovery of autoantibody-mediated encephalitides with variable clinical courses, we propose that the early diagnostic work-up should include evaluation for known paraneoplastic and autoimmune syndromes.

**Table 1:** Duration of prodromal and acute phases of Rasmussen’s encephalitis in the three cases.

	Prodromal (years)	Acute (years)
Case 1	1.5	3.5
Case 2	10	2
Case 3	2.5	3^a^

*^a^Indicates an approximation as this patient was lost to follow-up during the acute phase*.

MRI findings, specifically unihemispheric focal cortical atrophy, with or without T2 signal changes and caudate head hyperintensity or atrophy, are the second diagnostic criterion (18). While our three patients displayed some combination of these findings, again there was striking variability. Patients 1 and 3 showed marked hemiatrophy over time, whereas patient 2 has had much more focal volume loss. Though these differences may be accounted for by timing and duration of immunomodulatory therapy, variability of MRI findings, even rarely including bilateral changes, in both pediatric and adult populations appears to be the rule rather than the exception (10, 21–27). Dual pathology, which is concomitant cortical dysplasia with RE, is a particularly well documented phenomenon (28–35). Contrast enhancement of RE lesions, as seen in patient 3, has also been documented elsewhere (6). Furthermore, there is great diversity in the timing and duration of structural changes. Late-onset and rather slow changes have spurred clinicians to develop novel methods to detect more subtle changes in cortical volume (4, 36–39). Based on this multiplicity of MRI findings, we conclude that bilateral MRI findings and coexistent cortical dysplasia should not distract from a diagnosis of RE and advanced imaging analyzes should be performed when a diagnosis of RE is suspected.

EEG observations, specifically unihemispheric slowing with or without epileptiform activity and unilateral seizure onset, represent the third diagnostic criterion. Again, while all three patients in our case series fulfilled the above criterion at some point along their clinical course, the EEG showed great diversity and evolution over time. As noted by other investigators, EEG abnormalities are neither unihemispheric (16, 23), nor correlative with MRI changes (2, 22, 24, 40). Importantly, much as there is a natural history to the clinical course, EEG pathology also appears to evolve (8, 19, 41, 42). This is best evidenced by patients 1 and 3, who demonstrated both spread across the affected hemisphere as well as to the contralateral hemisphere (Figure 2). Notably, the background, or interictal EEG, of both patients also deteriorated over time (Figure 2).

Lastly, histopathology may be utilized as a final diagnostic criterion for RE. Inclusion criteria include T-cell dominant encephalitis with activated microglia, and exclusion criteria include the presence of numerous parenchymal macrophages, B cells, plasma cells, or viral inclusion bodies (18). Only one patient in our series underwent brain biopsy, which showed rare T cells. As discussed by Olson et al. (43), this may have been a false negative based on timing of biopsy. In particular, the prodromal and residual phases of the disease may not show a T-cell predominance (43). In addition, the specimen size may have been inadequate for diagnosis. A large neuropathologic study of 45 hemispherectomies showed that cerebral cortex pathology is multifocal, making diagnosis based on a focal resection difficult. That same study, although done in a pediatric population, also found that age of onset inversely correlated with severity of pathology (44), raising the idea of more subtle pathologic findings in adult-onset RE. Much like the MRI and EEG observations, the histopathology of RE appears more complex than the diagnostic criterion definition. Again, findings may be bilateral (45, 46) and multiple pathologies, namely cortical dysplasia (30–32, 47) may co-occur with RE. Given these observations, we suggest that concomitant pathology not distract from a diagnosis of RE. Furthermore, given the multifocal nature of RE pathology and potential absence of pathology in mild disease or during prodromal periods, we would not encourage the use of focal cortical biopsy to aid in RE diagnosis.

Our discussion to this point has focused on the diagnostic challenge of RE. Henceforth, we will discuss how these diagnostic characteristics have informed pathophysiology as well as treatment strategies. Histopathological findings have led to the guiding hypothesis that immune-mediated mechanisms are, in part or completely, responsible for disease progression. Studies have shown important roles for both cell-mediated and humoral immune responses; cytotoxic T cells (48, 49), gamma delta T cells (50), autoantibodies (21, 51, 52), microglia (53, 54), and astrocytes (55) have all been pathologically implicated. Newer literature has extended these findings to the level of the genome (54, 56). While immune-mediated mechanisms certainly have a pivotal role in disease progression, it is unlikely they represent the sole pathologic mechanism. Several authors have now proposed a multifactorial etiology of RE. Evidence pointing toward a shared-susceptibility network of autoimmune diseases bolsters this idea (57).

While there is currently no single accepted therapeutic option, the three mainstays of treatment are anti-epileptic drugs, immunomodulatory therapy, and surgical resection. Nearly universally, the seizures in RE have been refractory to medical therapy, as evidenced by our case series. Given the immune-mediated inflammatory findings on CSF analysis, MRI, and biopsy, various forms of immunomodulatory therapy have been trialed in RE. Immune therapies attempted in RE include high-dose steroids (58–60), intravenous immunoglobulin (7, 9, 58, 61–64), plasma exchange (65), azathioprine (57, 66), tacrolimus (11, 58, 61), natalizumab (66), and rituximab (67). A detailed discussion of these studies is beyond the scope of this paper and a near complete review may be found in Varadkar et al. (68). As might be expected *a priori* given the clinical variability of RE, response to immune therapy has ranged from slowing of progression (11, 59–61, 69) to essentially curative (7, 62, 63, 67). One fundamental concept of many immune-mediated encephalitides that appears to be true in RE as well is the benefit of early therapy (58, 69, 70). In our own case series, patient 2, who has been on the most consistent immune therapy (IVIG and Rituximab), has shown the least progression of disease. That said, just as in many other case reports and series, it is impossible to know if this is a therapeutic effect or a fundamental difference in the disease process in this patient. Nonetheless, we propose that clinicians consider a trial of immune therapy of a months-long duration in cases of progressive epilepsy with unknown etiology.

Lastly, surgical resections ranging from limited cortical resections (17) to lobectomies (20, 71) to hemispherectomies (24, 44) have been performed in RE. The vast majority of literature on this topic originates from the pediatric population, as the likelihood of permanent, disabling deficits is limited, compared to adults. Specifically, in the case of our patients, 2/3 had significant involvement of language centers, making hemispherectomy likely to leave patients not only hemiplegic but also globally aphasic. Moreover, as discussed earlier, late diagnosis after the presence of significant hemispheric deficits is a pitfall in adult populations and limits surgical considerations in cases where the static deficits, rather than the seizures are the major pathology. A broad discussion of various surgical techniques attempted is beyond the scope of this manuscript, but we would like to emphasize some potential surgical limitations. The multifocal pathologic findings, concomitant pathologies such as cortical dysplasia, contralateral MRI and EEG abnormalities, as well as a limited collective knowledge about newer immune therapies all potentially confound not only surgical planning but also post-surgical prognostication.

## Conclusion

This case series highlights not only the diagnostic and therapeutic challenges of adult-onset RE, but also brings to light the limitations of current diagnostic criteria. While adult-onset RE may be considered a variant, the overwhelming variability of this disease entity and the absence of a “typical” disease course may negate the need for such labeling. In relation to treatment strategies, large prospective trials are limited by the relative rarity of the disease, difficulty of early diagnosis thus limiting prompt therapy, and the lack of an acceptable biomarker for disease severity.

## Ethics Statement

This retrospective case series review was approved by the Montefiore Medical Center Institutional Review Board. As this was a case series review, consent was exempted.

## Author Contributions

JC and JM were responsible for data acquisition and interpretation, literature review, and manuscript preparation. FL provided guidance in data interpretation and aided in manuscript preparation.

## Conflict of Interest Statement

The authors declare that the research was conducted in the absence of any commercial or financial relationships that could be construed as a potential conflict of interest.
